# Colistin-Resistant *mcr-1*-Positive *Escherichia coli* ST131-*H*22 Carrying *bla*_CTX–M–15_ and *qnrB19* in Agricultural Soil

**DOI:** 10.3389/fmicb.2021.659900

**Published:** 2021-04-09

**Authors:** Ralf Lopes, João Pedro Rueda Furlan, Lucas David Rodrigues dos Santos, Inara Fernanda Lage Gallo, Eliana Guedes Stehling

**Affiliations:** Department of Clinical Analyses, Toxicology and Food Science, School of Pharmaceutical Sciences of Ribeirão Preto, University of São Paulo, Ribeirão Preto, Brazil

**Keywords:** acquired polymyxin resistance, emerging zoonotic *E. coli*, extended-spectrum β-lactamase, food and environmental safety, genomic surveillance, *mcr-1*, multidrug-resistant, One Health

## Abstract

The pandemic *Escherichia coli* sequence type 131 (ST131) carrying plasmid-mediated colistin resistance *mcr* genes has emerged worldwide causing extraintestinal infections, with lineages belonging to three major clades (A, B, and C). Clade B is the most prevalent in animals, contaminating associated meat products, and can be transmitted zoonotically. However, the *bla*_CTX–M–15_ gene has only been associated with C2 subclade so far. In this study, we performed a genomic investigation of an *E. coli* (strain S802) isolated from a kale crop in Brazil, which exhibited a multidrug-resistant (MDR) profile to clinically significant antimicrobials (i.e., polymyxin, broad-spectrum cephalosporins, aminoglycosides, and fluoroquinolones). Whole-genome sequencing analysis revealed that the S802 strain belonged to serotype O25:H4, ST131/CC131, phylogenetic group B2, and virotype D5. Furthermore, S802 carried the clade B-associated *fimH22* allele, genes encoding resistance to clinically important antimicrobials, metals, and biocides, and was phylogenetically related to human, avian, and swine ST131-*H*22 strains. Additionally, IncHI2-IncQ1, IncF [F2:A-:B1], and ColE1-like plasmids were identified harboring *mcr-1.1*, *bla*_CTX–M–15_, and *qnrB19*, respectively. The emergence of the *E. coli* ST131-*H*22 sublineage carrying *mcr-1.1*, *bla*_CTX–M–15_, and *qnrB19* in agricultural soil represents a threat to food and environmental safety. Therefore, a One Health approach to genomic surveillance studies is required to effectively detect and limit the spread of antimicrobial-resistant bacteria and their resistance genes.

## Introduction

The rapid spread of plasmid-mediated colistin resistance *mcr* genes has gained worldwide attention as a critical public health issue, since colistin is a last resort antimicrobial used to treat severe infections caused by multidrug-resistant (MDR) and extensively drug-resistant (XDR) bacteria (Perez et al., 2016; Tsuji et al., 2019).

Currently, epidemiological studies have shown that the spread of colistin-resistant *mcr*-positive bacteria is not a concern restricted to hospitals, but also represents a growing problem involving environmental and food safety. In this regard, various factors such as environmental sources, food-producing animals, international travel, and food trade, have accelerated the worldwide spread of *mcr*-type genes at the human-animal-environment interface (Liu et al., 2016; Hassan and Kassem, 2020; Johura et al., 2020).

In this context, the pandemic *Escherichia coli* sequence type 131 (ST131) carrying *mcr*-type genes has emerged causing extraintestinal infections (Liu et al., 2018; Mamani et al., 2019; Reid et al., 2019; Li et al., 2021). The complex subclonal structure of ST131 elucidated three major clades, each associated with a specific allele of the type 1 fimbrial adhesin gene (*fimH*), namely clade A with *fimH41*, clade B with *fimH22*, and clade C with *fimH30* (Petty et al., 2014; Stoesser et al., 2016).

Most studies have focused on the ST131-*H*30 sublineage, which is one of the leading causes of extraintestinal infections in humans, including C2 subclade associated with the *bla*_CTX–M–15_ gene (Dahbi et al., 2014; Matsumura et al., 2015; Mamani et al., 2019). In contrast, the most prevalent animal ST131 strains belong to the ST131-*H*22 sublineage and can be transmitted zoonotically, presenting a public health challenge (Liu et al., 2018; Roer et al., 2019; Saidenberg et al., 2020).

Specifically, contamination of crops by critical priority pathogens is of great concern, since these pathogens can also contaminate vegetables for consumption (Cantas et al., 2013; Araújo et al., 2017; Lopes et al., 2017; Reid et al., 2020; Lopes et al., 2021), increasing the risk of human exposure to antimicrobial-resistant bacteria, including *mcr*-positive strains. Despite this, little is known about the occurrence of bacteria carrying *mcr*-type genes in soils. Therefore, in this study, we performed a genomic investigation of an *mcr-1*-positive *E. coli* strain exhibiting an MDR profile to clinically significant antimicrobials and isolated from agricultural soil in the light of the One Health context that integrates human, animal, and environmental health.

## Materials and Methods

### Soil Sampling and Bacterial Isolation

During a surveillance study conducted between October and December 2019 to monitor the presence of clinically significant MDR Gram-negative bacteria in crops, 15 soil samples with a history of cow manure use were collected at a depth of ∼5 cm from chicory (*n* = 3), kale (*n* = 3), mustard (*n* = 3), parsley (*n* = 3), and chive (*n* = 3) crops on a farm in the state of São Paulo (21°00′36.0″ S; 47°27′00.0″ W), Brazil. All samples were stored at 4 °C and processed within 24 h. For bacterial isolation, 1 g of soil was inoculated in Luria-Bertani broth (Oxoid Ltd., United Kingdom) and incubated at 37 °C for 24 h. Subsequently, the cultures were streaked onto MacConkey agar plates (Oxoid Ltd., United Kingdom) supplemented with ceftriaxone (2 μg/ml) or colistin (2 μg/ml). Colonies were picked from the selective plates, subcultured, and streaked to obtain pure cultures. Bacterial identification was initially performed using 16S rRNA gene sequencing (Weisburg et al., 1991).

### Antimicrobial Susceptibility Testing and Detection of Resistance Genes

Antimicrobial susceptibility testing was performed by disk diffusion, VITEK 2 (biomérieux, France), and/or agar dilution methods with interpretative criteria from CLSI guidelines [CLSI (Clinical and Laboratory Standards Institute), 2020]. Colistin minimum inhibitory concentration (MIC) was determined by broth microdilution according to EUCAST^1^. Extended-spectrum β-lactamase (ESBL) production was screened by the double-disk synergy test (Jarlier et al., 1988). Additionally, *mcr-*type (*mcr-1* to *mcr-9*) and *bla*_CTX–M_-type (*bla*_CTX–M–1,_ *bla*_CTX–M–2,_ *bla*_CTX–M–8_, and *bla*_CTX–M–9_ groups) genes were investigated by PCR (Dallenne et al., 2010; Liu et al., 2016; Xavier et al., 2016; Borowiak et al., 2017; Carattoli et al., 2017; Yin et al., 2017; Yang et al., 2018; Wang et al., 2019).

### DNA Isolation and Whole-Genome Sequencing

For whole-genome sequencing (WGS), total DNA was extracted from an overnight culture using the GenElute^TM^ Bacterial Genomic DNA Kit (Sigma-Aldrich, United States) according to the manufacturer’s instructions. Sequencing was performed using the Illumina HiSeq 4000 (2 × 150 bp) platform (Illumina, United States).

### Data Processing, Assembly, and Genome Analysis

A quality check of the raw sequencing data was performed using the FastQC v.0.11.9 program^2^ and the reads were trimmed with Trimmomatic v.0.39 (Bolger et al., 2014). The quality value used for the base-calling program was Q = 20. In the next step, *de novo* genome assembly was carried out with SPAdes v.3.15.0 (Bankevich et al., 2012) and annotation was performed with Prokka v.1.14.5 (Seemann, 2014). Sequence type, serotype, FimH type, and clonotype were identified using MLST v2.0 (Larsen et al., 2012), SerotypeFinder v.2.0 (Joensen et al., 2015), FimTyper v.1.0 (Roer et al., 2017), and CHTyper v.1.0 (Roer et al., 2018), respectively. Antimicrobial resistance genes were detected using ResFinder v.4.1 (Zankari et al., 2012) and Antibiotic Resistance Gene-ANNOTation (ARG-ANNOT) v.4 (Gupta et al., 2014). Metals and biocides resistance genes were analyzed by BacMet v.2.0 (Pal et al., 2014). VirulenceFinder v.2.0 (Joensen et al., 2014) and the Virulence Factor Database (VFDB) v.R5 (Chen et al., 2005) were used to detect virulence genes, whereas virulence phylogroup was determined using the online Clermont typing tool^3^.

### Phylogenetic Analysis

For phylogenetic analysis, we selected the *E. coli* strain reported in this study and 849 other strains representative of all clades (A, B, and C) of *E. coli* ST131. A minimum spanning tree was constructed based on the MSTree v.2 algorithm and the wgMLST scheme in Enterobase^4^. The tree was visualized with iTOL v.5.7 (Letunic and Bork, 2019).

### Plasmid Assembly, Annotation, and Typing

Putative plasmid contigs were assembled using plasmidSPAdes v.3.15.0 (Antipov et al., 2016) and subjected to BLASTn analysis followed by gap closure. Annotation was performed by the Rapid Annotations using Subsystems Technology (RAST) server (Aziz et al., 2008) and manually curated with Geneious v.11.1.5 (Biomatters Ltd., Auckland, New Zealand). Plasmid replicon types and multilocus sequence typing were determined using PlasmidFinder v.2.1 and pMLST v.2.0 (Carattoli et al., 2014), respectively.

### Conjugation Assays

Conjugation assays were conducted using azide-resistant *E. coli* C600 as recipient strain. Overnight cultures of donor and recipient strains were mixed (ratio 1:1) and incubated for 18 h at 37 °C without shaking as previously described (Furlan et al., 2020a). Transconjugants were selected using MacConkey agar (Oxoid Ltd., United Kingdom) supplemented with sodium azide (200 μg/ml) and ceftriaxone (2 μg/ml), or sodium azide (200 μg/ml) and colistin (2 μg/ml), and confirmed by PCR for the detection of *mcr-* and *bla*_CTX–M_-type genes as described above.

## Results

### MDR *mcr-1*-Positive ESBL-Producing *E. coli* Isolated From Agricultural Soil

In this study, the presence of a *mcr-1*-positive ESBL-producing *E. coli* strain (named S802), identified by 16S rRNA gene sequence analysis and pairwise genome comparison of average nucleotide identity, was confirmed in one soil sample from the kale crop. In addition, the *E. coli* strain S802 displayed an MDR profile, defined as resistant to at least one antimicrobial of three or more different categories (Magiorakos et al., 2012). The MDR profile of *E. coli* S802 included resistance to colistin, penicillin, cephalosporins, aztreonam, aminoglycosides, quinolones, tetracycline, and chloramphenicol. In contrast, the strain displayed an intermediary resistance profile to ampicillin/sulbactam, remaining susceptible to piperacillin/tazobactam, amikacin, and carbapenems (Table 1).

**TABLE 1:** MICs of antimicrobials for *mcr-1*-positive ESBL-producing *E. coli* strain S802 from agricultural soil.

Antimicrobials	MIC (μg/ml)^a^
Ampicillin	**≥256**
Ampicillin/sulbactam	16/8
Piperacillin/tazobactam	2/4
Ceftazidime	**32**
Ceftriaxone	**≥256**
Cefotaxime	**≥256**
Cefepime	**32**
Aztreonam	**16**
Ertapenem	0,5
Imipenem	1
Meropenem	1
Gentamicin	**64**
Amikacin	2
Ciprofloxacin	**8**
Tetracycline	**≥256**
Chloramphenicol	**32**
Colistin	**4**

**^*a*^MIC values indicating resistance are shown in bold*.*

### Identification of the Pandemic *Escherichia coli* ST131 Lineage and Phylogenetic Analysis

**FIGURE 1:**
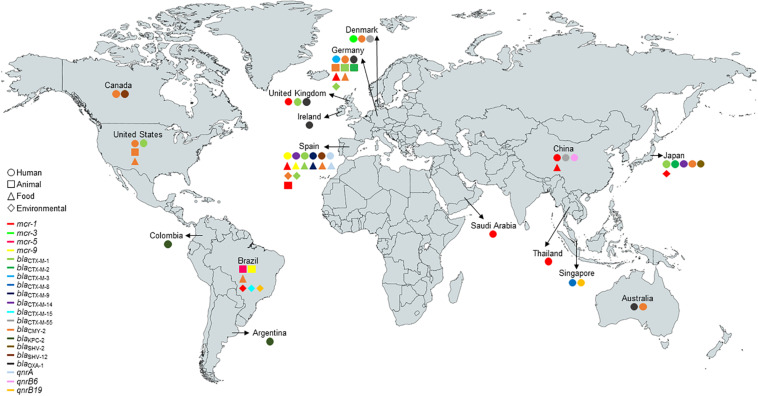
Worldwide distribution and sources of *E. coli* ST131-*H*22 carrying *mcr*-type, β-lactamase, and *qnr*-type genes at the human-animal-environment interface. The figure was produced using data retrieved from Enterobase (http://enterobase.warwick.ac.uk/species/index/ecoli) and Pubmed (https://pubmed.ncbi.nlm.nih.gov/) (*bla*_TEM_-types were not included in the figure).

WGS revealed that *E. coli* strain S802 belonged to serotype O25:H4 and phylogroup B2, known for including highly virulent extraintestinal lineages. Strain S802 carried *fimH22* allele and was assigned to the clade B pandemic ST131/CC131 lineage (Figure 1). In addition, the clonotype CH40-22 was determined.

**FIGURE 2:**
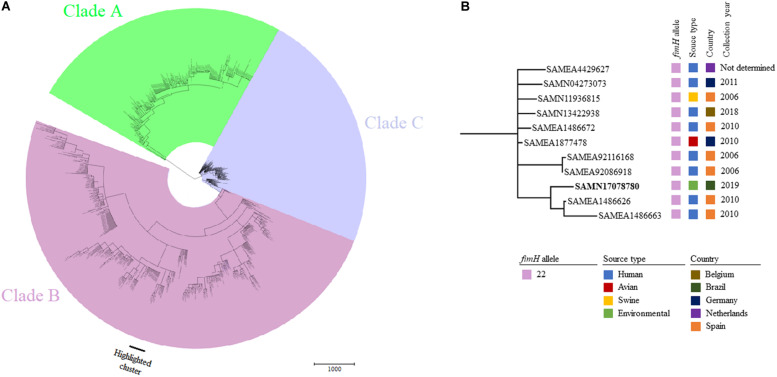
**(A)** Minimum spanning tree based on wgMLST of 850 worldwide distributed *E. coli* strains belonging to ST131. **(B)** Highlighted cluster containing *E. coli* S802 (BioSample accession indicated in bold) and showing the *fimH* allele, source of origin, country, and collection year of closely related strains.

Phylogenetic relatedness among 850 genomes of globally reported *E. coli* ST131 strains (Figure 2A) assigned S802 to a cluster comprising human *E. coli* ST131-*H*22 strains from Spain, Netherlands, Germany, and Belgium; one avian strain from Germany; and one swine strain from Spain. *E. coli* strain S802 was most related to two strains isolated from humans in Spain in 2010 (Figure 2B).

### Wide Resistome Against Multiple Antimicrobial Categories

In addition to the colistin resistance gene *mcr-1.1*, WGS analysis showed that ESBL production in the S802 strain was associated with the presence of the *bla*_CTX–M–15_ gene. Furthermore, a wide resistome was detected encoding other resistance determinants to β-lactams (*bla*_TEM–1A_, *bla*_TEM–1B_), aminoglycosides [*aac(3)-IIa*, *aadA1*, *aadA2b*, *aph(3′)-Ia*, *aph(3*″*)-Ib*, *aph(6)-Id*], fluoroquinolones (*qnrB19*), sulphonamides (*sul1*, *sul2*, *sul3*), trimethoprim (*dfrA1*, *dfrA5*), macrolides (*mdfA*), phenicols (*catA1*, *cmlA1*), and tetracyclines (*tetA*), as well as mutations in the quinolone resistance-determining region of *gyrA* (Ser83Leu, Asp87Asn) and *parC* (Ser80Ile) (Table 2).

**TABLE 2:** Genome features, resistome, and virulome of *E. coli* S802.

Feature^a^	Chromosome^b^	pS802-MCR	pS802-CTX-M	pS802-QnrB
Size (bp)	4,963,667 (94 contigs)	285,390	162,792	3,041
GC content (%)	50.47	48.47	52.48	52.09
No. of genes	4,688	315	174	2
Inc group (pMLST)	NA	HI2 (ST4)-Q1	F [F2:A-:B1]	ColE1-like
Resistome				
Polymyxins		*mcr-1.1*		
β-Lactams		*bla*_TEM–1A_	*bla*_CTX–M–15_, *bla*_TEM–1B_	
Aminoglycosides		*aac(3)-IIa*, *aadA1*, *aadA2b*, *aph(3*″*)-Ib*, *aph(6)-Id*	*aph(3′)-Ia*, *aph(3*″*)-Ib*, *aph(6)-Id*	
Quinolones				*qnrB19*
Sulphonamides		*sul1*, *sul2*, *sul3*	*sul2*	
Trimethoprim		*dfrA1*	*dfrA5*	
Macrolides	*mdfA*			
Phenicols		*catA1*, *cmlA1*		
Tetracyclines		*tetA*	*tetA*	
Metals	*corAB, cueOR*, *cutACEF*, *cusSRCFBA, nikABCDER*, *pcoCD*, *rcnABR*, *ruvB*, *tehAB*, *zraP*	*merRTPCADE*, *pcoEABCDRSE*, *silESRCBAP*, *terY3Y2XY1W*, *terZABCDEF*	*merRTPCADE*	
Biocides	*acrAEFS*, *cpxA, emrABEKRY*, *evgAS*, *fetAB*, *gadCEWX*, *marRAB*, *mdtABCEFGHKNOP sugE*, *tehAB*, *tolC*	*qacEΔ1*	*sitABCD*	
QRDR mutations	*gyrA* (Ser83Leu, Asp87Asn), *parC* (Ser80Ile)			
Virulome	*chuA*, *cnf1*, *fyuA*, *gad*, *hlyCABD*, *hra*, *ibeA, irp1*, *irp2, kpsE*, *kpsM II-K5*, *ompT*, *papIBAHCDJKEFG*, *usp*, *ybtAEPQSTUX*, *yfcV*		*cia*, *cvaABC/cvi*, *etsC*, *hlyF*, *iroBCDEN*, *iss*, *iucABCD*, *iutA*, *mchF*, *ompT*, *sitABCD*, *traT*	

**^*a*^QRDR, Quinolone resistance-determining region. *^*b*^NA, Not applicable*.**

Genes predicted to confer tolerance to metals, including copper (*cueOR*, *cutACEF*, *pcoEABCDRSE*), silver (*silESRCBAP*), copper/silver (*cusSRCFBA*), mercury (*merRTPCADE*), tellurium (*tehAB*, *terY3Y2XY1W*, *terZABCDE*), tellurium/selenium/chromium (*ruvB*), nickel (*nikABCDE*), nickel/cobalt/iron (*rcnABR*), cobalt/magnesium/manganese (*corAB*), and zinc (*zraP*) were also identified.

Regarding biocides resistance, genes encoding efflux pumps, transport modulators, and other proteins associated with resistance to acridines (*acrAEFS*, *tehAB*, *tolC*), chlorhexidine (*cpxA*), crystal violet (*mdtABCEFGHKNOP*, *tehAB*), ethidium bromide (*acrAEFS*, *sugE*, *tehAB*, *tolC*), hydrochloric acid (*gadCEWX*), hydrogen peroxide (*cpxA*, *fetAB*, *sitABCD*), organic solvents (*marRAB*), quaternary ammonium compounds (*acrAEFS*, *cpxA, emrABEKRY*, *mdtABCEFGHKNOP sugE*, *tolC*), sodium deoxycholate (*evgAS*), and sodium dodecyl sulfate (*acrAEFS*, *emrABEKRY*, *mdtABCEFGHKNOP sugE*, *tolC*) were detected (Table 2).

### Virulome

Virulome analysis of *E. coli* S802 revealed a diversity of virulence factors, including *chuA* (outer membrane hemin receptor), *cia* (colicin Ia), *cnf1* (cytotoxic necrotizing factor), *cvaABC/cvi* [colicin (microcin) V operon], *etsC* (putative type I secretion outer membrane protein), *fyuA* (yersiniabactin receptor), *gad* (glutamate decarboxylase), *hlyCABD* (α-hemolysin operon), *hlyF* (hemolysin F), *hra* (heat-resistant agglutinin), *ibeA* (invasin of brain endothelial cells), *iroBCDEN* (salmochelin operon), *irp1-2*/*ybtAEPQSTUX* (yersiniabactin synthesis), *iss* (increased serum survival lipoprotein), *iucABCD*/*iutA* (aerobactin operon), *kpsE* (capsule polysaccharide export inner-membrane protein), *kpsM II-K5* (polysialic acid transport protein; group II capsule), *mchF* (ABC transporter protein), *ompT* [outer membrane protease (protease 7)], *papIBAHCDJKEFG* (P fimbriae operon), *sitABCD* (iron and manganese transport system), *traT* (complement resistance protein), *usp* (uropathogenic specific protein), and *yfcV* (fimbrial protein) (Table 2). In this regard, *E. coli* S802 was assigned to the virotype D5, based on the presence of the *cnf1*, *hlyA*, *ibeA*, *kpsM II-K5*, and *papGIII* genes (Dahbi et al., 2014).

### Plasmids and Horizontal Transfer

**FIGURE 3:**
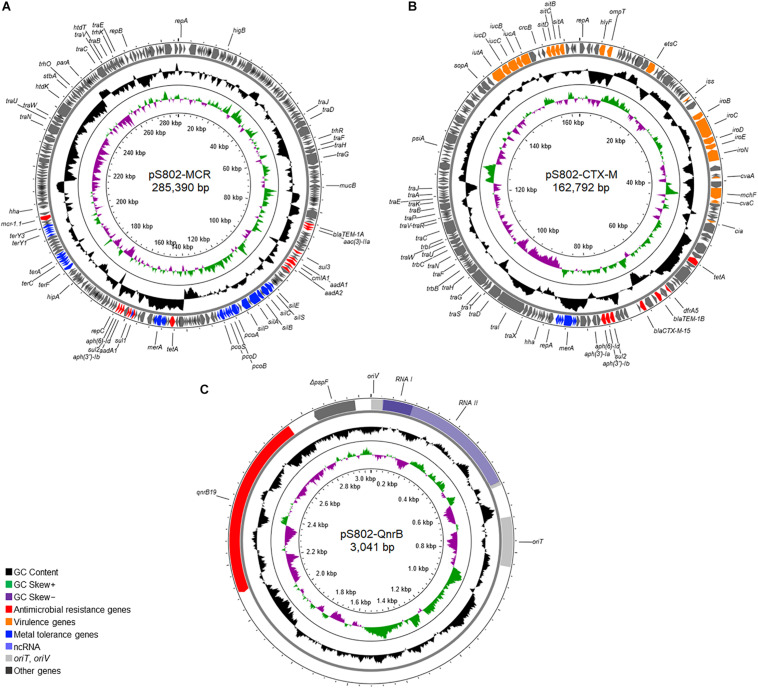
Overview of the plasmids **(A)** pS802-MCR, **(B)** pS802-CTX-M, and **(C)** pS802-QnrB carrying *mcr-1.1*, *bla*_CTX–M–15_, and *qnrB19*, respectively, in *E. coli* S802.

Three plasmids, named pS802-MCR, pS802-CTX-M, and pS802-QnrB, were harbored by the S802 strain and carried *mcr-1.1*, *bla*_CTX–M–15_, and *qnrB19*, respectively (Figure 3).

**FIGURE 4:**
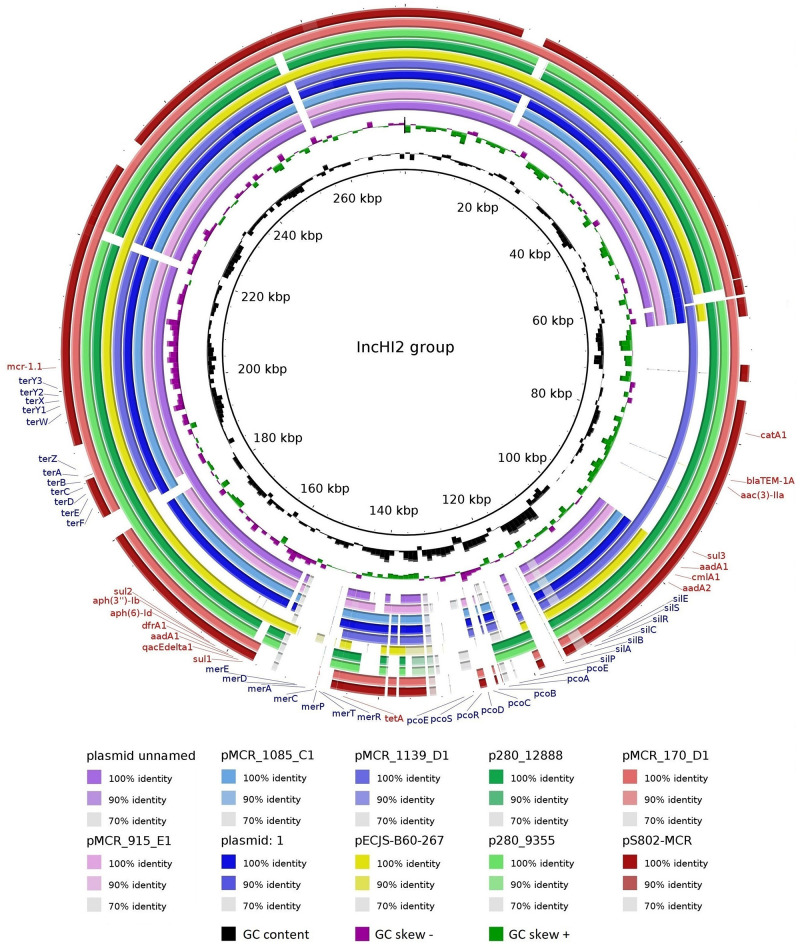
Comparison between IncHI2 plasmids. Plasmids of *E. coli* strains: plasmid unnamed (GenBank: CP015833.1) from a human in the United Kingdom; plasmid: 1 (GenBank: LR882919.1) from a human in the Netherlands; pECJS-B60-267 (GenBank: KX254341.1) from a pig in China; pS802-MCR (GenBank: MW495059.1) from soil in Brazil; pMCR_915_E1 (GenBank: MT929285.1), pMCR_1085_C1 (GenBank: MT929286.1), pMCR_1139_D1 (GenBank: MT929287.1), and pMCR_170_D1 (GenBank: MT929288.1) from turkey meat in the Czech Republic. Plasmids of *Salmonella* Schwarzengrund strains: p280_12888 (GenBank: CP045449.1) and p280_9355 (GenBank: CP045446.1) from poultry in Brazil. Matches with less than 70% identity and no matches appear as blank spaces. Resistance genes to antimicrobials/biocides and metals are indicated in red and blue, respectively.

The pS802-MCR plasmid was 285,390 bp in length, containing 48.47% GC and 315 coding regions (CDSs), and belonged to the HI2 (ST4) and Q1 incompatibility groups. Besides *mcr-1.1*, this plasmid carried *bla*_TEM–1A_, *aac(3)-IIa*, *aadA1*, *aadA2b*, *aph(3*″*)-Ib*, *aph(6)-Id*, *sul1*, *sul2*, *sul3*, *dfrA1*, *catA1*, *cmlA1*, *tetA*, *merRTPCADE*, *pcoEABCDRSE*, *silESRCBAP*, *terY3Y2XY1W*, *terZABCDEF*, and *qacEΔ1* resistance genes (Table 2). Analysis of the genetic context of *mcr-1.1* revealed the presence of IS*Apl1* upstream and *pap2* downstream of the gene. Moreover, pS802-MCR showed a high nucleotide identity with other IncHI2 plasmids of *E. coli* strains isolated from animal, human, and food in European and Asian countries, as well as with plasmids of *Salmonella* Schwarzengrund strains isolated from poultry in Brazil (Figure 4).

**FIGURE 5:**
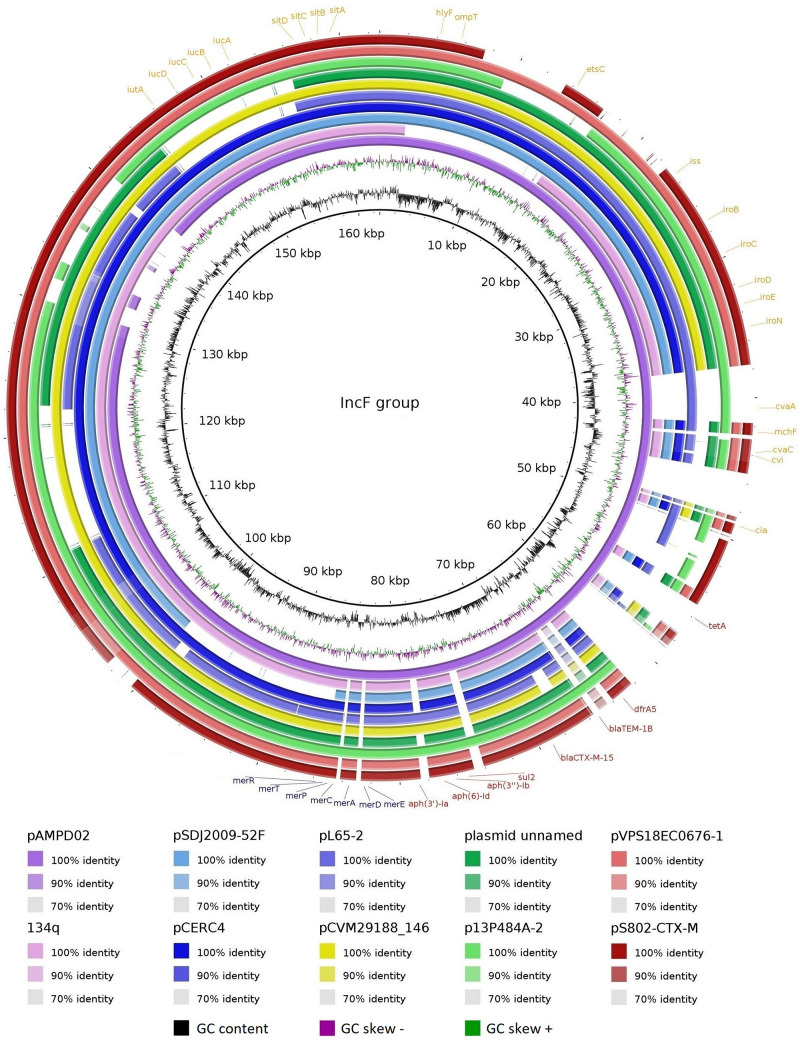
Comparison between IncF plasmids. Plasmids of *E. coli* strains: pAMPD02 (GenBank: CP058310.1) from a giant panda in China; 134q (GenBank: CP023363.1) from a canine in Scotland; pSDJ2009-52F (GenBank: MH195200.1) and pCERC4 (GenBank: KU578032.1) from a human in Australia; plasmid unnamed (GenBank: CP027485.1) from a human in the United States; pL65-2 (GenBank: CP034739.1) from a goose in China; p13P484A-2 (GenBank: CP019282.1) from a pig in China; pVPS18EC0676-1 (GenBank: CP063726.1) from veal in the United States; pS802-CTX-M (GenBank: MW495061.1) from soil in Brazil. Plasmid of *Salmonella* Kentucky: pCVM29188_146 (GenBank: CP001122.1) from poultry in the United States. Matches with less than 70% identity and no matches appear as blank spaces. Resistance genes to antimicrobials and metals are indicated in red and blue, respectively, whereas virulence genes are indicated in orange.

The pS802-CTX-M plasmid was a 162,792 bp IncF [F2:A-:B1] plasmid, containing 52.48% GC and 174 CDSs. In addition to the *bla*_TEM–1B_, *aph(3′)-Ia*, *aph(3*″*)-Ib*, *aph(6)-Id*, *sul2*, *dfrA5*, *tetA*, *merRTPCADE*, and *sitABCD* resistance genes (Table 2), the ISE*cp1*-*bla*_CTX–M–15_-Δ*orf477* transposition unit was identified inserted in the transposon Tn*2* truncated by IS*26* in this plasmid. Virulence genes, namely *cia*, *cvaABC/cvi*, *etsC*, *hlyF*, *iroBCDEN*, *iss*, *iucABCD*, *iutA*, *mchF*, *ompT*, *sitABCD*, and *traT*, were also carried on pS802-CTX-M. The pS802-CTX-M plasmid exhibited a high nucleotide identity with IncF plasmids of *E. coli* strains isolated from animal, human, and food in North American, Asian, Australian, and European countries (Figure 5).

Additionally, pS802-MCR and pS802-CTX-M harbored genes involved in the replication (*rep* genes), partition/maintenance (*par* genes), conjugation (*tra*, *trb*, *trh* operons), toxin-antitoxin systems (*higB/higA*, *hipB/hipA*, *hok/sok*, *relE/parE*), and inhibition of SOS response (*psiAB*). Conjugation assays confirmed transfer of pS802-MCR and pS802-CTX-M from *E. coli* S802 at frequencies of 4.25 × 10^–6^ and 5.32 × 10^–3^ transconjugants/receptor, respectively.

The pS802-QnrB was a small 3,041 bp ColE1-like plasmid, containing 52.09% GC and only the *qnrB19* and Δ*pspF* (truncated transcription activator) genes (Table 2). The *qnrB19* gene was located in the conserved genetic context between the sequence encoding the regulatory RNAII and the Xer-mediated recombination site. The pS802-QnrB plasmid was related to other ColE1-like plasmids of Enterobacterales isolated worldwide at the human-animal-environment interface and shared 70% query coverage and ∼99.5% nucleotide identity with plasmids of the same incompatibility group of *E. coli* strains isolated from poultry in Brazil (GenBank accession numbers: KX452393.1 and KX452394.1), similarly to the IncHI2-IncQ1 plasmid in this study.

## Discussion

The emergence of clinically relevant bacterial strains in soils is an underestimated public and environmental health problem that requires attention. In this regard, *mcr*-positive *E. coli* lineages from farming soil and agricultural soil have been previously reported in China (Zheng et al., 2017) and Algeria (Touati et al., 2020), respectively. In Brazil, *mcr*-type genes from soil samples have only been detected in total DNA or cultivable microbiota so far (Oliveira et al., 2019; Furlan et al., 2020b; Dos Santos et al., 2020). In this study, we report for the first time the presence of an *mcr-1*-positive *E. coli* isolated from the soil ecosystem in American countries, representing a potential risk of human exposure to antimicrobial-resistant bacteria.

*E. coli* belonging to the ST131 pandemic high-risk clone has been identified in human, animal, environmental, and food samples (Figure 1). In addition, *E. coli* ST131 has been frequently reported carrying clinically significant antimicrobial resistance genes, such as *mcr*-types and/or ESBL genes (Rodrigues et al., 2017; Reid et al., 2019), and associated with extraintestinal diseases, mainly bloodstream and urinary tract infections (Liu et al., 2018; Mamani et al., 2019; Reid et al., 2019).

Whereas *E. coli* ST131-*H*30 is the most prevalent sublineage causing extraintestinal infections in humans (Dahbi et al., 2014; Matsumura et al., 2015; Mamani et al., 2019), ST131-*H*22 predominates in animals, contaminating associated meat products, and can be transmitted zoonotically (Liu et al., 2018; Roer et al., 2019; Saidenberg et al., 2020). Findings from our phylogenetic analysis showed that avian, swine, and human ST131-*H*22 strains were closely related, supporting results from previous studies (Liu et al., 2018; Reid et al., 2019; Roer et al., 2019; Saidenberg et al., 2020), and also included our environmental strain in the same cluster as those strains (Figure 2), highlighting their transmission at the human-animal-environment interface.

Notably, IncF [F1:A2:B20] plasmids without *bla*_CTX–M–15,_ the most clinically relevant ESBL gene worldwide (Bevan et al., 2017), and IncF [F2:A1:B-] plasmids with this gene are the most frequently associated with the C1 and C2 subclades of ST131, respectively (Johnson et al., 2016; Pitout and DeVinney, 2017). In contrast, IncF [F2:A-:B1] without *bla*_CTX–M–15_ is commonly detected in clade B (Reid et al., 2019; Flament-Simon et al., 2020). Interestingly, we reported the presence of *bla*_CTX–M–15_ in clade B of ST131 in this study (Figure 1). Analysis of pS802-CTX-M, an IncF [F2:A-:B1] plasmid, revealed that the ISE*cp1*-*bla*_CTX–M–15_-Δ*orf477* transposition unit was inserted in a truncated Tn*2* transposon, highlighting the role of the insertion sequence IS*Ecp1* for the mobilization of *bla*_CTX–M–15_ onto plasmids (Dhanji et al., 2011; Zong et al., 2015).

Additionally, pS802-CTX-M harbored the ColV region, frequently identified in avian pathogenic *E. coli* (APEC) and associated with increased fitness and virulence of these strains (Johnson et al., 2006). The presence of ColV plasmid in *E. coli* strains isolated from humans can indicate evidence of zoonotic transmission (Rodriguez-Siek et al., 2005; Liu et al., 2018). As detected in the present study, ColV plasmids can also carry multiple antimicrobial resistance genes, which is clinically relevant due to the combination of virulence and resistance determinants in a single mobile genetic element (Flament-Simon et al., 2020).

Although the origin of the *E. coli* ST131-*H*22 high-risk sublineage carrying the *mcr-1.1*, *bla*_CTX–M–15_, and *qnrB19* genes was not investigated, cow manure used for soil fertilization was the most likely source. In addition, other animal (e.g., wild animal feces), human (e.g., sewage), and environmental (e.g., contaminated irrigation water) sources could be involved in the dissemination of clinically relevant bacterial strains (Beuchat, 2002; Cantas et al., 2013; Araújo et al., 2017).

The range of hosts and sources of the *E. coli* ST131-*H*22 sublineage, including soil detected here, supports a genetic versatility and adaptation mediated by the gene content, which includes genes encoding resistance to antimicrobials, biocides, and heavy metals. In fact, the plasmids pS802-MCR and pS802-CTX-M co-harboring resistance genes to antimicrobials, biocides, and heavy metals were identified (Figures 3–5). In this regard, heavy metals could come from sources such as contaminated irrigation water, inorganic fertilizers, and pesticides commonly used in agricultural practices, remaining in the environment for long periods (Gimeno-Garcia et al., 1996; Sipter et al., 2008; Osaili et al., 2016; Bhilwadikar et al., 2019). Consequently, these compounds, as well as biocides, may act as selectors of strains resistant to antimicrobials.

Finally, the presence of MDR pathogens displaying a broad resistome in agricultural soil could lead to contamination of vegetables and, since these foods are usually consumed raw, the risk of human exposure to antimicrobial-resistant bacteria with clinical interest increases (Reid et al., 2020; Lopes et al., 2021). Although ingestion of these bacteria may not immediately have a direct impact on health, colonization by this pathway can contribute to the horizontal gene transfer of antimicrobial resistance to the gut microbiome (Maeusli et al., 2020). Thereafter, a potential threat to human health would be associated with future endogenous infections, mainly in immunosuppressed patients, in whom therapeutic failure could occur.

## Conclusion

The emergence of zoonosis-associated *E. coli* ST131-*H*22 carrying a broad resistome, including *mcr-1.1*, *bla*_CTX–M–15_, and *qnrB19*, in agricultural soil represents a potential risk of human and animal exposure to antimicrobial-resistant bacteria and/or their resistance genes, posing a threat to public and environmental health. Also considering the possible contamination of vegetables for consumption from soil pathogens, appropriate measures, such as the improvement of agricultural practices, in addition to stricter regulations, need to be taken. Therefore, a One Health approach is required to effectively limit the spread of MDR bacteria and prevent their health impacts.

## Data Availability Statement

The datasets presented in this study can be found in online repositories. The names of the repository/repositories and accession number(s) can be found below: https://www. ncbi.nlm.nih.gov/genbank/, JAENHI000000000.1, https://www. ncbi.nlm.nih.gov/genbank/, MW495059.1, https://www.ncbi.nlm.nih.gov/genbank/, MW495060.1, https://www.ncbi.nlm.nih.gov/genbank/, MW495061.1.

## Author Contributions

RL, JF, LS, and IG carried out the research. RL and JF performed data curation and formal analysis. RL, JF, and ES conceived and designed the study, and reviewed and edited the manuscript. RL drafted the original manuscript. ES coordinated and acquired funding for the study. All authors read and approved the final manuscript.

## Conflict of Interest

The authors declare that the research was conducted in the absence of any commercial or financial relationships that could be construed as a potential conflict of interest. The reviewer MD declared a shared affiliation with the authors to the handling editor at the time of review.

## Footnotes

1 www.eucast.org

2 http://www.bioinformatics.babraham.ac.uk/projects/fastqc/

3 http://clermontyping.iame-research.center/

4 https://enterobase.warwick.ac.uk/species/index/ecoli
